# Supplementary material for: Evaluation of Selected Quality Parameters of “Agristigna” Monovarietal Extra Virgin Olive Oil and Its Apple Vinegar-Based Dressing during Storage
Source: Foods. 2022 Apr 13;11(8):1113. doi: 10.3390/foods11081113 (PMC9024682; doi:10.3390/foods11081113)
Supplement: Supplementary file 1 [file foods-11-01113-s001.zip › foods-1651845-supplementary.pdf]

Supplementary

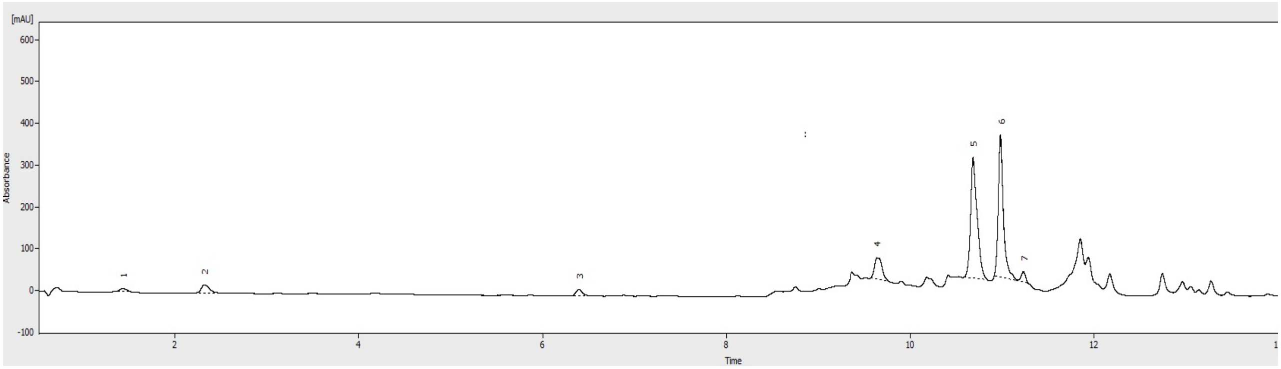

Figure S1. UHPLC chromatograms of Agristigna EVOO. Identified compounds: (1) hydroxytyrosol; (2) tyrosol; (3) p-cumaric acid; (4) oleuropein; (5) luteolin; (6) pinoresinol; (7) apigenin.
